# O035: Third federal state wide survey on MRSA management in North Rhine-Westphalian hospitals

**DOI:** 10.1186/2047-2994-2-S1-O35

**Published:** 2013-06-20

**Authors:** A Jurke, R Köck, AW Friedrich, R Kämmerer, I Daniels-Haardt

**Affiliations:** 1 Infectiology and Hygiene, NRW Centre of Health, Germany; 2 Institute of Hygiene, University Hospital Münster, Münster, Germany; 3 Dept. of Medical Microbiology and Infection Prevention, University Medical Center Groningen, Groningen, The Netherlands; 4 North Rhine Westphalian Ministery of Health, Emancipation, Care and Age, Düsseldorf, Germany; 5 Health Protection, Health Reporting, NRW Centre of Health, Münster, Germany

## Introduction

*Staphylococcus aureus* (*S. aureus*) is a major cause of healthcare-associated infections. In Germany in 2011, about 18.2% of *S. aureus* from blood cultures were methicillin-resistant (MRSA).

## Objectives

In 2011, the Ministry of Health, Emancipation, Care and Age of North Rhine-Westphalia (NRW), initiated the third federal state wide survey in hospitals to inspect the MRSA-management and implementation of the recommendations of the German national Commission for Hospital Hygiene and Infection Control (KRINKO).

## Methods

All hospitals were requested to submit the number of MRSA cases per 1,000 patient-days, the number of colonisations or infections, stratified in imported or nosocomial, the proportion of MRSA isolates in all *S. aureus* isolates from blood cultures and the number of blood culture samples taken in the year 2011. In addition, the implementation of the KRINKO recommendations in the hospitals has been assessed by local health authorities.

## Results

The response rate was 97.8%; 92.7% of the 315 hospitals provided analyzable data.

The mean MRSA incidence density was 2.59 per 1,000 patient days; the median was 1.35 with quartiles of 0.89 and 1.99. In 254 hospitals a mean of 21.0% (median 18.0%, quartiles of 5.9 and 27.0) of all *S. aureus* detected in blood cultures were MRSA. The hospitals screened in average 21.1% (median 12.0, lower and upper quartiles of 4.8 and 29.7.) of all patients on admission for the carriage of MRSA. The local health authorities appraised, that 85% of the responding hospitals have been adequately addressed the national health recommendations.

## Conclusion

The data give insight in MRSA prevalence and management of hospitals providing service to 18 million inhabitants. The study has led to a greater awareness about MRSA in regional hospitals and revealed the progress achieved since 2006. Since 2006 the mean screening rate has nearly quadrupled and the mean MRSA incidence density increased by 36%. The implementation of the KRINKO recommendations must be improved further as well as the data quality. The survey is a pragmatic instrument to monitor the MRSA prevention and control measures in hospitals in a federal state.

## Disclosure of interest

None declared.

